# The Impact of Malnutrition Risk and Perioperative Complications in Gastrointestinal Cancer Patients Undergoing Elective Major Surgery: A Prospective Observational Multicenter Study

**DOI:** 10.3390/nu18020325

**Published:** 2026-01-20

**Authors:** Manuel Durán-Poveda, Gil Rodríguez Caravaca, Alejandro Suárez-de-la-Rica, Diego Rodríguez Villar, Andrés Sánchez Pernaute, Emilia Cancer Minchot, Julia Ocón Bretón, Tamara Díaz-Vico, Brezo Martínez-Amores

**Affiliations:** 1 Department of Digestive and General Surgery, Hospital Universitario Rey Juan Carlos, 28933 Madrid, Spain; manuel.duran@hospitalreyjuancarlos.es; 2 Department of Medical Specialties and Public Health, Faculty of Health Sciences, Hospital Universitario Rey Juan Carlos, 28933 Madrid, Spain; gil.rodriguez@salud.madrid.org (G.R.C.); brezo.martinez@hospitalreyjuancarlos.es (B.M.-A.); 3 Service of Preventive Medicine, Hospital Universitario Fundación Alcorcón, 28922 Madrid, Spain; 4 Department of Anesthesiology and Surgical Care, Hospital Universitario de La Princesa, 28006 Madrid, Spain; alejandro.suarez.delarica@gmail.com; 5 Facultad de Ciencias Biomédicas y de la Salud, Universidad Alfonso X el Sabio (UAX), 28691 Madrid, Spain; 6 Department of Anesthesiology, Hospital Ramón y Cajal, 28034 Madrid, Spain; diego_rv_95@hotmail.com; 7 Department of Digestive and General Surgery, Hospital Clínico San Carlos, 28040 Madrid, Spain; pernaute@yahoo.es; 8 Service of Endocrinology and Nutrition, Hospital Universitario de Fuenlabrada, 28942 Madrid, Spain; 9 Service of Endocrinology and Nutrition, Hospital Universitario “Lozano Blesa”, 50009 Zaragoza, Spain; mjocon@salud.aragon.es; 10 Department of Medical Oncology, Hospital Universitario Rey Juan Carlos, 28933 Madrid, Spain

**Keywords:** gastrointestinal neoplasms, postoperative complications, nutritional status

## Abstract

**Background/Objectives:** The study aimed to characterize perioperative complications and their relationship with nutritional risk in gastrointestinal cancer patients undergoing surgical treatment. **Methods:** An observational, prospective, and multicenter study was carried out in 469 patients with gastrointestinal malignancies undergoing elective major abdominal surgical procedures in public hospitals throughout Spain. Complications developed during hospitalization and at 30 days after surgery were recorded, and the patients’ nutritional status was evaluated using the MUST screening tool. **Results:** Colorectal and gastric cancer were the most common tumors. Complications during hospitalization occurred in 146 patients (rate 31.1%). Infections accounted for 68.5% of complications, in particular surgical site infections (SSIs), followed by paralytic ileus (40.4%). At 30 days, the complication rate was 9%, with infections as the most common events. In patients with severe nutritional risk at discharge (MUST score ≥ 2), the percentage of patients with complications was 24.7% as compared to 9.2% in patients without complications (*p* < 0.0001). **Conclusions:** Clinicians should be aware of the high frequency of SSIs and that complications are higher among patients with severe nutritional risk. These findings emphasize the need for routine nutritional screening and targeted perioperative support in cancer patients undergoing gastrointestinal cancer surgery.

## 1. Introduction

Patients undergoing any surgical procedure, particularly major surgery, are at risk of developing perioperative complications, which can lead to increased morbidity and mortality, as well as increased length of hospital stay, healthcare costs, and prolonged recovery [1,2,3]. It has been estimated that about 234 million major surgical procedures are performed every year worldwide [4], and according to a prospective international 7-day cohort study of outcomes following elective adult inpatient surgery in 27 countries, 16.8% of patients developed one or more postoperative complications [5]. Also, in a 7-day cohort study of postoperative mortality across 498 hospitals from 28 European countries, an in-hospital mortality rate of 4% for elective surgery was reported [6]. Moreover, the aging of the population results in significant increases in the demand for surgical services [7], and elderly patients have a higher rate of major perioperative complications and mortality and a longer length of stay [5,8], which is further complicated by procedure-specific and patient-associated risk factors.

However, despite advances in anesthetic methods and surgical techniques with the widespread introduction of minimally invasive surgery and principles of enhanced recovery after surgery (ERAS), control of perioperative complications and subsequent improvement of outcome in surgical patients remains an important challenge in daily practice [9,10]. Risk assessment strategies have become a mandatory part of continuous quality improvement in the care of surgical patients.

Among specific patient-related risk factors for perioperative outcome, malnourished surgical patients experience higher postoperative mortality, morbidity, length of stay, and hospital readmission rates [11,12,13]. Malnutrition has been recognized as a poor prognostic factor in many surgical conditions, but it is particularly relevant in surgical cancer patients. In a retrospective nationwide analysis from 2005 to 2015 of 1,335,681 patients undergoing major cancer surgery, protein–energy malnutrition showed an estimated annual percentage increase of 7.2% and was significantly associated with increased risk of mortality, major complications, and higher total costs [14]. Likewise, in a study of 5709 colorectal and gastric cancer patients undergoing elective surgery from 381 hospitals in 75 countries, severe malnutrition was present in 33.3% of patients and was associated with an increased risk of 30-day mortality across all country income groups [15]. In colorectal cancer patients, and depending on the instrument used for the assessment of nutritional status, the rates of malnutrition varied between 20% and 37% [16,17].

Although it has been extensively recognized that nutritional screening is a key strategy for surgical patients, particularly for those with solid cancers, a systematic approach to addressing malnutrition of all patients at admission, followed by a detailed assessment of the nutritional status of patients at risk, is not fully implemented. Different screening tools for malnutrition in cancer patients are available, such as the Nutrition Risk Screening 2002 (NRS-2002), Malnutrition Screening Tool (MST), Mini-Nutrition Assessment (MNA), Malnutrition Universal Screening Tool (MUST), the Global Leadership Initiative on Malnutrition (GLIM), or the Patient-Generated Subjective Global Assessment (PG-SGA). The MUST score combines body mass index (BMI), unplanned weight loss, and disease effect and is a simple and easy screening instrument extensively used in hospitalized patients. The GLIM criteria also include practical phenotypic indicators (low BMI, unintentional weight loss, and reduced muscle mass), allowing for the categorization of the severity grade of malnutrition into moderate and severe. Both MUST and GLIM screening tools are widely used for the diagnosis of malnutrition in clinical practice. In a previous study of our group carried out in a cohort of 469 gastrointestinal cancer patients undergoing elective major surgical procedures, the rates of moderate and severe nutritional risk on admission using the Malnutrition Universal Screening Tool (MUST) were 17.9% and 21.1%, respectively [18]. Moreover, using the Global Leadership Initiative on Malnutrition (GLIM) criteria in patients with severe nutritional risk, moderate malnutrition was present in 35.3% of patients and severe malnutrition in 64.6%. An interesting finding of the study was that 47% of patients with severe nutritional risk on admission were also at severe risk at discharge, whereas 20.7% of patients without nutritional risk on admission had moderate/severe risk at discharge [18]. However, the characteristics of perioperative complications were not analyzed.

Therefore, a post hoc analysis was conducted to characterize perioperative complications and their relationship with nutritional risk in this cohort of adult patients undergoing elective major abdominal surgical procedures for the treatment of gastrointestinal cancer. Better knowledge of the relationships between perioperative complications and nutritional risk, nutritional support, and inclusion in an ERAS program is indispensable to promptly recognizing and managing potentially severe and life-threatening complications associated with poor nutritional status in cancer surgery patients.

## 2. Materials and Methods

### 2.1. Study Design and Participants

The PREMAS project (PREvalence of Malnutrition in gastrointestinal Surgical oncology Patients) was an observational, prospective, and multicenter study, with the participation of 23 public hospitals throughout Spain, in which major surgical procedures for cancer patients are performed on a routine basis. The main objective of the PREMAS study was to determine the prevalence of nutritional risk based on the MUST score in patients with gastrointestinal cancer undergoing elective surgical treatment. Results of the primary objective have been published previously [18]. The present post hoc analysis aimed to characterize complications developed during hospitalization and at 30 days after surgery, as well as the relationship between perioperative complications and the patient’s nutritional status. Briefly, inclusion criteria were adult patients (18 years or older) diagnosed with solid malignant tumors of the gastrointestinal tract scheduled for elective major surgery as primary cancer treatment at participating hospitals, hospital admission at least 48 h before surgery, and expected length of hospitalization of at least 5 days. Exclusion criteria were urgent surgery, minor surgical procedures as the reason for admission, and the presence of secondary malignant tumors of the gastrointestinal tract.

The study was conducted in agreement with the Declaration of Helsinki, and the study protocol was approved by the Ethics Committee of Hospital Clínico San Carlos (registration number 20/121-E, date 25 February 2020). Written informed consent was obtained from all patients.

### 2.2. Variables and Data Collection

Nutritional assessment was performed before surgery (within 48 h preoperatively) once the patient had been admitted to the hospital and at the time of hospital discharge using the MUST [19] and GLIM [20] screening tools. Briefly, MUST is a five-step scoring system based on measurements of body mass index (BMI), unplanned weight loss in the past 3–6 months, and the effect of acute disease, according to which the risk of malnutrition is classified into “low risk” (score 0), “medium risk” (malnourished) (score 1), and “severe risk” (score ≥ 2). The GLIM score was calculated in the group of patients with severe nutritional risk scoring ≥ 2 in the MUST screening tool. According to the GLIM instrument, moderate malnutrition was defined as unintended weight loss of 5–10% < 6 months or 10–20% > 6 months, low BMI < 20 kg/m^2^ if <70 years or < 22 kg/m^2^ if >70 years, and mild-to-moderate reduced muscle mass. Severe malnutrition was defined as unintended weight loss of >10% < 6 months or > 20% > 6 months, low BMI < 18.5 kg/m^2^ if <70 years or <20 kg/m^2^ if >70 years, and severe reduced muscle mass. Complications were evaluated using the Clavien–Dindo grade system classification, which is a uniform system for reporting negative surgical outcomes based on the degree of medical care required to achieve resolution. They are graded from I (minor complications) to V (death of the patient) [21,22].

In all patients, the following data were recorded: age; gender; race; civil status; living conditions; educational level; working status; place of residence; smoking habits; weight; height; BMI; location of gastrointestinal cancer; TNM classification; type of surgical procedure; comorbidities; the Charlson comorbidity index [23] (0–1 points: absence of comorbidity, 2 points: low comorbidity, ≥3 points: high comorbidity); the Barthel index for activities of daily living [24]; MUST and GLIM scores on admission; nutritional treatment during hospitalization; inclusion in ERAS program of the Spanish Group of Multimodal Rehabilitation (GERM) [25]; length of hospital stay; perioperative complications during hospitalization and at 30 days after surgery; Clavien–Dindo grade of complications; need of hospital readmission and reoperation due to complications.

### 2.3. Statistical Analysis

Categorical variables are expressed as frequencies and percentages, and continuous variables as mean and standard deviation (SD). The distribution of the study variables according to the presence or absence of complications was analyzed with the chi-square test for categorical variables and Student’s *t*-test for continuous variables. We also used Fisher’s exact test or the Mann–Whitney *U* test, according to the conditions of application. Statistical significance was set at *p* ≤ 0.05. Variables with a *p*-value < 0.2 in the bivariate analysis were included in a stepwise logistic regression model to identify risk factors for complications. The odds ratio (OR) and 95% confidence interval (CI) were calculated. The Statistical Analysis System (SAS Institute, Cary, NC, USA) version 9.4 was used for data analysis.

## 3. Results

### 3.1. Characteristics of the Patients

The study population included 469 patients (62% men), with a mean (SD) age of 68.2 (11.7) years (range 23–93 years) and BMI of 26.7 (4.3) kg/m^2^. The general characteristics of the study population are shown in Table 1. Most patients (71%) were married or had a partner and lived with their partner or family (85.9%). More than half (56.3%) had primary education, and 60.6% were retired, and 27.9% lived in cities with more than 500,000 inhabitants. Also, only 14.9% of patients were current smokers. Cardiovascular disease was the most frequent comorbidity (57.8%), and the mean Charlson comorbidity index was 2.9 (1.8). The mean Barthel index was 97.0 (9.4) with 82.5% of patients being totally independent.

In relation to nutritional status on admission, the mean MUST score was 0.7 (1.1). Nutritional risk was absent in most patients (61.0%) (MUST score 0). In the remaining 49% of patients at risk of malnutrition, moderate risk (MUST score 1) was present in 17.9% and severe risk (MUST score ≥ 2) in 21.1% (Table 1). Moreover, 35 patients (35.3%) met the GLIM criteria for moderate malnutrition, and 64 (64.6%) for severe malnutrition (Table 1).

Tumor- and surgery-related characteristics are shown in Table 2. Cancer of the colon was the most frequent malignancy (53.9%), followed by rectal cancer (23.7%), gastric cancer (8.1%), pancreatic cancer (5.3%), and esophageal cancer (3.6%). Surgical procedures for colorectal cancer (hemicolectomy, sigmoidectomy, low anterior rectal resection, and abdominoperineal resection) were also the most common operations. A small percentage of patients underwent gastrectomy (8.5%), esophagectomy (3.2%), and other procedures. Pancreatectomy was performed in 6.0% of patients.

Of the 469 patients included in the study, nutritional support at the time of admission to the hospital was recorded in 158 (33.7%). Also, during the period of hospitalization, 158 patients (33.7%) (the same number of patients) received nutritional treatment (mostly oral supplements). A total of 197 patients (42.0%) were included in an ERAS program. The mean length of stay in the intensive care unit (ICU) was 0.8 (3.9) days, and the mean length of hospital stay was 10.2 (9.8) days. None of the patients died.

### 3.2. Complications During Hospitalization

Complications during the inpatient stay occurred in 146 of the 469 patients, with a complication rate of 31.1%. As shown in Table 3, all infections and paralytic ileus were the most frequent complications. Infections accounted for 68.5% of complications, in particular surgical site infections (SSIs), both incisional and organ/space, with a rate of 47.3%. Other infections included urinary tract infection (8.2%), catheter-related infection (6.8%), and pneumonia (6.2%). Paralytic ileus was recorded in 40.4% of cases. Other less frequent complications were wound dehiscence, acute renal failure, intestinal occlusion, arrhythmia, deep vein thrombosis, and pulmonary edema. One patient with cardiac arrest was successfully resuscitated without neurological sequelae.

The majority of these complications were minor complications (20.6% were Clavien–Dindo grade I and 49.3% were Clavien–Dindo grade II). Grade III complications were observed in 41 patients (28.1%) and grade IVa in 3 (2.0%). Of the 41 patients with grade III complications, surgical interventions were performed in 25 (61%). The three cases of grade IVa included one patient with cardiac arrest, one patient with bleeding, and another with pulmonary edema.

### 3.3. Complications at 30 Days After Surgery

Complications recorded 30 days after the surgical procedure occurred in 44 patients of 469 patients, with a 30-day complication rate of 9.4%. The characteristics of complications are shown in Table 3. Infections were also the most common events (88.6%) of the cases, especially SSIs (especially incisional). Paralytic ileus and wound dehiscence each accounted for 11.4% of complications. Other events, such as acute renal failure, arrhythmia, cardiogenic edema, or deep vein thrombosis, were uncommon.

**Figure 1:**
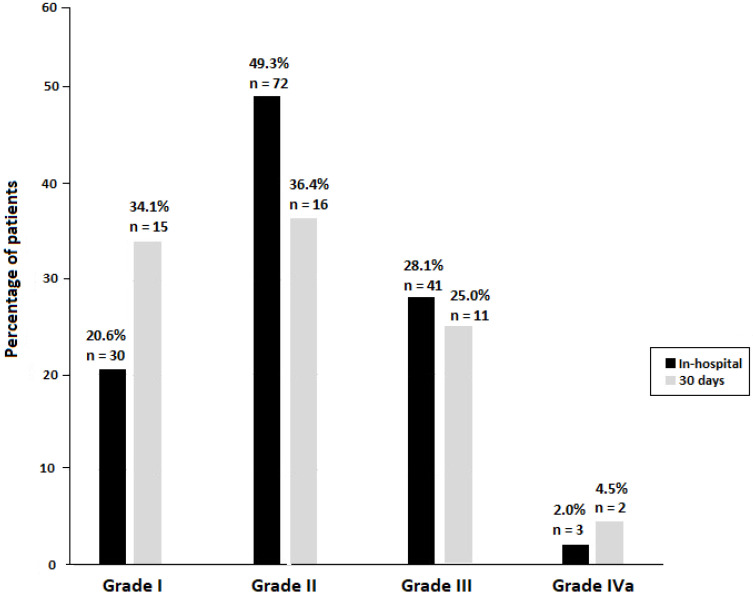
Complications during hospital stay (*n* = 144) and at 30 days after surgery (*n* = 44) according to Clavien–Dindo grade in 469 gastrointestinal cancer patients undergoing elective surgery.

Also, 70.5% of complications were Clavien–Dindo grade I (34.1%) or grade II (36.4%). Grade III complications were registered in 11 (25%) patients and grade IVa in 2 (4.5%) patients. Eleven patients required readmission to the hospital, with a readmission rate of 2.3%, and three patients were reoperated. The comparison of Clavien–Dindo grades of complications occurring both during hospitalization and at 30 days after surgery is shown in Figure 1. In both time periods, minor complications were the most commonly reported.

### 3.4. Complications and Nutritional Status

As shown in Table 4, there were significant differences in nutritional risk at discharge according to the presence of complications, with lower percentages of patients with MUST score 0 and higher percentages with MUST score ≥ 2 among those with complications (*p* = 0.0001). Differences in complication rates according to GLIM criteria were not found. Also, the percentage of patients with complications was lower in participants of an ERAS program than in those who did not participate (38.4% vs. 61.6%, *p* = 0.361). Moreover, the percentage of patients who received nutritional support during hospitalization was lower among those with complications (46.7%) than among those without complications (53.3%) (*p* = 0.0001). Data on complications in patients with MUST score ≥ 1 at the time of hospital admission are shown in Table S1 (Supplementary Material).

The MUST score at hospital discharge in all 146 patients with complications during hospitalization was 0.9 (1.2), but it was significantly higher among those who received nutritional treatment (*n* = 105) as compared to those without nutritional support (*n* = 41) (1.1 [1.2] vs. 0.5 [0.9], *p* = 0.012).

Results of multivariable analysis are shown in Table 5. Independent variables significantly associated with moderate–severe malnutrition at hospital discharge were nutritional risk on hospital admission (MUST score ≥ 1) and the presence of complications and nutritional treatment during hospitalization.

## 4. Discussion

The present findings in a large prospective cohort of gastrointestinal cancer patients undergoing major surgical procedures for the treatment of their primary solid tumors in Spain contribute to better characterizing perioperative complications, which would help clinicians improve patients’ care in this surgical setting [26,27]. Complex gastrointestinal surgical procedures with curative intent in cancer patients, including radical colorectal, esophageal, gastric, pancreatic, or hepatic resections, are associated with high complication rates, as well as comorbidity and mortality. Also, a wide range of risk factors are amenable to actions in perioperative care and prehabilitation programs, which may lead to improved outcomes, especially for high-risk patients [28].

In the present study, postoperative complications during hospitalization were recorded in 31.1% of the patients. This high rate is consistent with data reported in other clinical series of patients with gastrointestinal cancer undergoing extensive surgical resections, with rates ranging between 12.5% to 51% across gastric carcinoma patients [29], over 40% in colorectal cancer patients [27], about 59% after esophagectomy [30], or up to 54% for pancreatic resection [31]. In relation to the type of complications, infections and paralytic ileus were the most common events, followed by wound dehiscence, bleeding, and acute renal dysfunction. Infectious complications occurred in 68.5% of the cases, with SSIs, both incisional and organ/space, accounting for 69% of all infections. Although a high percentage of SSI resolve with adequate measures, including prompt antibiotic treatment and early surgical wound debridement, a systematic review of the burden of SSIs based on 26 studies from six European countries showed that SSIs as compared with uninfected patients were consistently associated with elevated costs, prolonged hospitalization, reoperation, and readmission, and that SSIs increased mortality rates [32]. Therefore, proper assessment of patient-related and surgical-related risk factors for SSIs is essential for preventing SSIs. According to the Surgical Infection Division of the Spanish Association of Surgery [33], the best measures with the highest degree of evidence to be applied for the prevention of SSIs are avoiding removal or clipping of hair from the surgical field, skin decontamination with alcohol-based solutions, adequate systemic antibiotic prophylaxis (administration within 30–60 min before the incision in a single preoperative dose; intraoperative re-dosing when indicated), maintenance of normothermia and perioperative maintenance of glucose levels.

The rate of 40.4% of paralytic ileus found in our study, which is somewhat higher than rates of 10–30% depending on the procedure and reported in the literature [34], may be explained by the relatively old age of the population and the large percentage of colorectal cancer surgeries. Other complications, such as deep vein thrombosis, intestinal occlusion, or arrhythmia, were uncommon. It is remarkable that 69.9% of complications occurring during hospitalization were Clavien–Dindo grades I and II, but among the patients with grades III and IVa, all were successfully treated, and none of the patients died. Complications recorded after 30 days of surgery when the patients had already discharged from the hospital followed the same pattern as early complications, with infections (88.6%) (SSIs 71.8%), paralytic ileus, dehiscence, hemorrhage, and acute renal failure as the most frequent. Other complications, such as deep vein thrombosis, pulmonary edema, and eviscerations, were more common than those recorded in the immediate postoperative period. Clavien–Dindo grade IVa complications occurred in two patients with pulmonary edema.

Interestingly, the mean MUST score at hospital discharge in the patients with complications was 0.9 (1.2), which indicates a low–medium nutritional risk. However, the percentage of patients with severe nutritional risk at discharge, that is, with a MUST score ≥ 2, was significantly higher among patients with complications as compared with the uncomplicated group. Moreover, in the logistic regression analysis, the presence of complications was an independent predictor of moderate–severe nutritional risk at discharge. On the other hand, the inclusion in an ERAS program was associated with a lower percentage of patients with complications.

An interesting observation among the 146 patients with complications was that the percentage of those receiving nutritional support during hospitalization with moderate/severe nutritional risk (MUST score ≥ 2) at discharge was significantly higher than that of those who did not receive nutritional support. Likewise, patients treated with nutritional support showed a higher mean MUST score at discharge than patients who did not receive nutritional support. These findings obtained in the group of 146 patients with complications are consistent with data previously reported for the entire cohort of 469 gastrointestinal cancer patients, in which 43% of patients who received nutritional therapy continued to have moderate/severe nutritional risk on discharge, and 54% of those with MUST score ≥ 2 on admission maintained this score at discharge [18]. Therefore, improvements in nutritional therapy during hospitalization are a crucial factor for preventing an increase in nutritional risk, which is especially important in patients with esophageal, gastric, pancreatic, and small intestine cancer tumors. Clinicians should also be aware of the nutritional needs of gastrointestinal cancer patients because almost one-fourth of patients were at severe nutritional risk (MUST score ≥ 2), and 64.6% of them met GLIM criteria of severe malnutrition at the time of hospital admission. Also, it should be noted that among patients with moderate risk of malnutrition (MUST ≥ 1) at the time of hospital admission, inclusion in an ERAS program or nutritional support during hospitalization did not appear to reduce the rate of complications.

## 5. Conclusions

In the present prospective cohort study of gastrointestinal cancer patients undergoing elective surgical treatment, the rate of complications during hospitalization was 31.1% and 9% at 30 days after surgery. Infections, especially SSIs, accounted for the highest percentage of complications, which should alert clinicians to be aware of this type of complication and to establish intraoperative and postoperative measures, including prompt antibiotic treatment and early surgical wound debridement when necessary. Complications were significantly higher among patients with severe nutritional risk, which reinforces the need for intensive nutritional support for this subset of patients, as well as for implementing protocols for systematic screening of nutritional status in gastrointestinal cancer patients undergoing extensive radical surgical procedures.

## Figures and Tables

**Table 1:** General characteristics of gastrointestinal cancer patients undergoing elective surgery on admission to the hospital.

Variables	Total Patients (*n* = 469) N (%)
**Gender**	
Men	291 (62.0)
Women	178 (38.0)
**Age, years, mean (SD)**	68.2 (11.7)
**Civil status**	
Single	45 (9.6)
Married/partner	333 (71.0)
Widower	69 (14.7)
Separated/divorced	22 (4.7)
**Living conditions**	
Alone	63 (13.4)
Partner or family	403 (86.0)
Nursing home	3 (0.6)
**Educational level**	
Primary education	264 (56.3)
Secondary education	153 (32.6)
University level	52 (11.1)
**Working status**	
Active/student/housewife	123 (26.2)
Retired	284 (60.6)
Sick leave/unemployed	62 (13.2)
**Place of residence**	
500,000 to >1 million	131 (28.0)
200,001–500,000	101 (21.5)
50,000–200,000	94 (20.0)
<50,000	143 (30.5)
**Smoking habit**	
Smoker	70 (15.0)
Ex-smoker	177 (37.7)
Never smoker	222 (47.3)
**Body mass index (BMI), kg/m^2^, mean (SD)**	26.7 (4.3)
**Comorbidity** (multi-response)	
Cardiovascular disease	271 (57.8)
Diabetes mellitus	110 (23.4)
Chronic obstructive pulmonary disease (COPD)	51 (10.9)
Other	178 (38.0)
**Concomitant treatment** (multi-response)	
Chemotherapy neoadjuvant	108 (23.0)
Radiotherapy neoadjuvant	72 (15.3)
Chronic immunosuppressants (at least 3 months)	6 (1.3)
Chronic corticoids (equivalent doses of 5 mg prednisone for at least 3 weeks)	4 (0.8)
**Charlson comorbidity index, mean (SD)**	2.9 (1.8)
**Barthel index score, mean (SD)**	97.0 (9.4)
<20 (total dependency)	2 (0.4)
21–60 (severe dependency)	4 (0.9)
61–90 (moderate dependency)	48 (10.2)
91–99 (sight dependency)	28 (6.0)
100 (independent)	387 (82.5)
**Nutritional risk**	
MUST score, mean (SD)	0.7 (1.1)
MUST score 0 (no risk)	286 (61.0)
MUST score 1 (moderate risk)	84 (17.9)
MUST score 2 (severe risk)	99 (21.1)
GLIM criteria for moderate malnutrition	35 (35.3)
GLIM criteria for severe malnutrition	64 (64.6)

Data expressed as numbers and percentages in parentheses unless otherwise stated. SD: standard deviation; MUST: Malnutrition Universal Screening Tool; GLIM: Global Leadership Initiative on Malnutrition.

**Table 2:** Tumor localizations and surgical procedures of gastrointestinal cancer patients undergoing elective surgery.

Variables	Total Patients (*n* = 469) N (%)
**Primary tumor localization**	
Colon	253 (53.9)
Rectum	111 (23.7)
Stomach	38 (8.1)
Pancreas	25 (5.3)
Esophagus	17 (3.6)
Small bowel	8 (1.7)
Liver	5 (1.1)
Biliary tree	4 (0.8)
Missing	8 (1.8)
**Surgical procedure** (multi-response)	
Right hemicolectomy	128 (27.3)
Left hemicolectomy/Sigmoidectomy	122 (26.0)
Low anterior rectal resection	87 (18.5)
Gastrectomy	40 (8.5)
Abdominoperineal resection	30 (6.4)
Pancreatectomy	28 (6.0)
Hepatectomy	19 (4.0)
Esophagectomy	15 (3.2)
Total colectomy	9 (1.9)

**Table 3:** Complications in gastrointestinal cancer patients undergoing elective surgery.

Variables	Complications	
	During Hospitalization Total Patients (*n* = 146) N (%)	30 Days After Surgery Total Patients (*n* = 44) N (%)
**Infections**	100 (68.5)	39 (88.6)
Surgical site infection (organ/space)	41 (28.1)	11 (25.0)
Surgical site infection (incisional)	28 (19.2)	17 (38.6)
Urinary tract infection	12 (8.2)	6 (13.6)
Catheter-related infection	10 (6.8)	1 (2.3)
Pneumonia	9 (6.2)	4 (9.1)
**Paralytic ileus**	59 (40.4)	5 (11.4)
**Wound dehiscence**	25 (17.1)	5 (11.4)
**Hemorrhage (gastrointestinal/other)**	25 (17.1)	6 (13.6)
**Acute renal failure**	22 (15.1)	4 (9.1)
**Arrhythmia**	6 (4.1)	2 (4.5)
**Cardiogenic pulmonary edema**	4 (2.7)	3 (6.8)
**Intestinal occlusion**	4 (2.7)	1 (2.3)
**Deep vein thrombosis**	2 (1.4)	2 (4.6)
**Evisceration**	2 (1.4)	1 (2.3)
**Cardiac arrest**	1 (0.7)	0
**Pulmonary embolism**	0	1 (2.3)

**Table 4:** Relationship between in-hospital complications and nutritional risk at hospital discharge.

Variables	Complications During Hospitalization		
	Yes, *n* (%)	No, *n* (%)	*p*-Value
**MUST score at hospital discharge**			
0 (*n* = 304)	75 (24.7)	229 (75.3)	0.0001
1 (*n* = 93)	35 (37.6)	58 (62.4)	
≥2 (*n* = 65)	36 (55.4)	29 (44.6)	
**GLIM criteria**			
Moderate malnutrition (*n* = 32)	18 (56.2)	14 (43.7)	0.278
Severe malnutrition (*n* = 63)	27 (42.8)	36 (57.1)	
**Inclusion in an ERAS program**			
Yes (*n* = 193)	56 (38.4)	137 (43.3)	0.361
No (*n* = 269)	90 (61.6)	179 (56.6)	
**Inpatient nutritional support**			
Yes (*n* = 225)	105 (46.7)	120 (53.3)	0.0001
No (*n* = 237)	41 (17.3)	196 (82.7)	
Missing (*n* = 7)			

ERAS: Enhanced recovery after surgery.

**Table 5:** Risk factors for moderate–severe malnutrition at hospital discharge.

Variables	Odds Ratio (95% Confidence Interval)	*p*-Value
MUST score ≥ 1 vs. 0 on hospital admission	4.31 (2.80–6.62)	<0.001
Complications during hospitalization, yes vs. no	2.33 (1.48–3.66)	0.0003
Nutritional support during hospitalization, yes vs. no	2.08 (1.33–3.24)	0.001

## Data Availability

The data presented in this study are available on request from the corresponding author due to institutional policy.

## Supplementary Materials

The following supporting information can be downloaded at: https://www.mdpi.com/article/10.3390/nu18020325/s1. Table S1: Complications in patients with MUST score ≥ 1 at the time of hospital admission.

## Appendix A

**PREMAS Study Group:** Aitana García Tejero, Hospital San Pedro, Logroño; Héctor Guadalajara and Javier Barambio, Fundación Jiménez Díaz, Madrid; Gabriela Romay Cousido, Hospital Universitario de A Coruña, A Coruña; Inmaculada Monjero Ares, Hospital Lucus Agusti, Lugo; Héctor Marín Ortega, Hospital Universitario de Cruces, Barakaldo, Bilbao; Carmelo Loinaz Segurola, Hospital Universitario 12 de Octubre, Madrid: Emilio Peña, Hospital Reina Sofía, Murcia; Omar Abdellah, Hospital Universitario de Salamanca, Salamanca; Natalia González Alcolea, Isabel Prieto, Hospital Universitario La Paz, Madrid; Elisabet Julià i Verdaguer, Hospital Universitario Joan XXIII, Tarragona; Pedro Antonio Cascales, Hospital Universitario Virgen de la Arrixaca, Murcia; Cristóbal Zaragoza Fernández, Consorcio Hospital General Universitario de Valencia, Valencia; Antonio Arroyo Sebastián, Hospital General Universitario de Elche, Elche, Alicante; Virgilio Ruíz Luque, Hospital Universitario de Valme, Sevilla; Estíbaliz Gutiérrez Cafranga, Hospital de Jerez, Jerez, Cádiz; Lluis Oms, Ladislao Cayetano Paniagua, Consorci Sanitàri de Terrassa, Terrassa, Barcelona; Manuel Durán, Tamara Díaz, Brezo Martínez-Amores, Leire Zaraín, Hospital Universitario Rey Juan Carlos, Madrid; Elena Ruiz Ucar, Hospital Universitario de Fuenlabrada, Madrid; Manuela Elia, Hospital Universitario “Lozano Blesa”, Zaragoza; Andrés Sánchez Pernaute, Hospital Clínico San Carlos, Madrid; Carmen Balagué, Hospital de la Santa Creu i Sant Pau, Barcelona; Miguel Camblor, Hospital Universitario Gregorio Marañón, Madrid; Jesús Alberto de la Rosa Báez, Hospital Universitario Juan Ramón Jiménez, Huelva.
