# Supplementary material for: The Impact of Malnutrition Risk and Perioperative Complications in Gastrointestinal Cancer Patients Undergoing Elective Major Surgery: A Prospective Observational Multicenter Study
Source: Nutrients. 2026 Jan 20;18(2):325. doi: 10.3390/nu18020325 (PMC12844973; doi:10.3390/nu18020325)
Supplement: Supplementary file 1 [file nutrients-18-00325-s001.zip › nutrients-4016919-supplementary.pdf]

Supplementary Material

Table S1. Complications in patients with MUST ≥ 1 at the time of hospital admission

Variables	Complications N (%)	*P* value
Total patients	183 (100)	
Evaluable patients	179 (97.8)	
ERAS program		
Yes (n = 76)	24 (31.6)	0.275
No (n = 103)	41 (39.8)	
Nutritional support on hospital admission		
Yes (n = 65)	35 (53.8)	0.277
No (n = 114)	51 (44.7)	
Nutritional support during hospitalization		
Yes (n = 65)	37 (56.9)	0.042
No (n = 114)	46 (40.3)	
